# Basal cell carcinoma—a clinical indicator of immunosuppression

**DOI:** 10.3389/fmed.2024.1381492

**Published:** 2024-03-14

**Authors:** Lucian G. Scurtu, Marian Petrica, Francesca Scurtu, Anca Angela Simionescu, Marco I. Popescu, Olga Simionescu

**Affiliations:** ^1^Department of Dermatology I, Colentina Hospital, “Carol Davila” University of Medicine and Pharmacy, Bucharest, Romania; ^2^“Gheorghe Mihoc-Caius Iacob” Institute of Mathematical Statistics and Applied Mathematics of the Romanian Academy, Bucharest, Romania; ^3^Faculty of Mathematics and Computer Science, University of Bucharest, Bucharest, Romania; ^4^Department of Obstetrics and Gynecology, Filantropia Clinical Hospital, “Carol Davila” University of Medicine and Pharmacy, Bucharest, Romania; ^5^Faculty of Medicine, “Titu Maiorescu” University, Bucharest, Romania

**Keywords:** basal cell carcinoma, immunosuppression, skin cancer, diabetes mellitus, cancer, carcinoma

## Abstract

**Background:**

Basal cell carcinoma (BCC) and squamous cell carcinoma (SCC) are skin-derived carcinomas. The literature strongly connects SCC with acquired immunosuppression. Current data regarding BCC’s association with immunosuppressive comorbidities are vague. The primary objective of this study was to establish the correlations between BCC and immunosuppressive comorbidities of patients. Materials and methods: We conducted a retrospective cohort study on 275 patients with a histopathological proven diagnosis of BCC from October 2019 to October 2023. Demographic data, BCC characteristics, and patients’ comorbidities were analyzed. Comorbidities were classified as non-immunosuppressant and immunosuppressant (primary and secondary immunodeficiencies).

**Results:**

We recorded 292 BCCs from 275 patients (142 females, 133 males), with equally distributed skin phototypes. 66.44% of the BCCs were detected in patients with various comorbidities (*p* < 0.001), of which 81.44% had immunosuppressive comorbidities (p < 0.001). All the immunosuppressive comorbidities were secondary and included diabetes mellitus (47.55%), history of solid or hematogenous cancer in the last 5 years (26.57%), chronic kidney disease (8.39%), chronic infections (9.09%), and antirheumatic immunosuppressive therapies (8.39%) (*p* < 0.001). BCC patients with immunosuppressive comorbidities did not develop larger BCCs (*p* = 0.2577) or more aggressive subtypes (*p* = 0.4269) and BCC did not arise earlier in their life (*p* < 0.001). BCC on the nasal pyramid was frequent in cancer history patients (*p* = 0.008). The ulcerated form of BCC is more confined to patients with chronic kidney disease (*p* = 0.006). Multiple BCCs are more frequent in patients with secondary immunodeficiencies (*p* = 0.027).

**Conclusion:**

BCC represents a clinical indicator of secondary immunodeficiency. Further research should establish if cancer screening campaigns may be beneficial in BCC patients.

## 1 Introduction

Basal cell carcinoma (BCC) is a non-melanoma skin cancer (NMSC) and represents the most common neoplasm in humans. The lifetime risk of developing a BCC is 20–30% and the incidence rates are predicted to continue to grow at least for the following 15 years. Due to its indolent behavior, the cancer registries do not collect data regarding BCC (1, 2). Age is an independent risk factor for BCC, even if some BCCs may arise early in life. The incidence of BCC doubles from 40 to 70 years of age. Men have higher BCC rates (1.5:1), but distribution does not vary among genders in young patients (2). Most of the BCCs arise secondary to UVB exposure, with more than 75% of cases with DNA mutations-cyclobutane dimer formation (UV signature mutation) and C>T transitions at pyrimidines sites. Hedgehog signaling pathway constitutive activation is displayed in most BCCs. This pathway regulates cell type differentiation and proliferation and regulates the cell cycle (3–5).

Based on the recurrence risk, basal cell carcinoma is classified into low-risk (superficial, nodular, pigmented) and high-risk (morpheiform/ infiltrative, basosquamous, micronodular, and ulcerated) subtypes (6, 7). Chronic sun exposure is a well-established environmental risk factor for developing BCC, alongside intermittent sun exposure (2). Other acknowledged risk factors are fair skin phototypes, artificial tanning, arsenic exposure, ionizing radiation, ultraviolet A light phototherapy, and immunosuppression in organ transplant recipients (OTRs) (2). Surgical excision represents the gold-standard treatment for BCC. Other therapies include destructive treatments and the new emerging therapies for advanced BCC: the Hedgehog pathway inhibitors (vismodegib, patidegib, taladegib, sonidegib) and cemiplimab. Vismodegib and sonidegib are currently approved for patients with locally advanced BCC ineligible for radiation therapy or surgery (8, 9).

OTRs have an increased risk of developing keratinocyte cancer: BCC incidence is 10 times higher, and cutaneous squamous cell carcinoma (cSCC) incidence is 250 times higher than the general population. BCC does not display aggressive behavior in OTRs, unlike cSCC. OTRs develop cSCC at a younger age than the general population (10, 11). The relationship of cSCC to immunosuppression is generally understood (11), and that of BCC is still being elucidated among researchers.

In the last few decades, BCC has been highly studied in OTRs. However, organ-transplant-associated immunosuppression does not stand as the most frequent cause of impaired immunocompetence. While primary immunodeficiencies consist of rare, inherited conditions, secondary immunodeficiencies are more prevalent in daily practice and generally include chronic viral infections, solid and hematogenous malignancies, chronic kidney disease (CKD), diabetes mellitus (DM), asplenia, end-stage heart failure and treatment with immunosuppressive drugs (including biologics and long-term systemic steroids) (12, 13).

The main objective of this study is to establish the correlations between BCC and immunosuppressive comorbidities of patients. An additional purpose is to characterize patients’ demographic data and BCC characteristics (size, localization, histopathological subtypes) in a southeastern European population.

## 2 Methods

### 2.1 Study design

This research represents a cross-sectional, retrospective study conducted at two academic centers from “Carol Davila” University of Medicine and Pharmacy, Bucharest, Romania. The study was conducted on patients with confirmed BCC diagnosis over 4 years (from October 2019 through October 2023). STROBE guidelines for cross-sectional studies were followed.

### 2.2 Study population and data collection

Electronic medical records were reviewed for patients with a biopsy-proven BCC diagnosis and included the following variables: sex, age at diagnosis, living environment, skin phototype, patient detailed history, tumor size, localization, and histopathological subtypes. Patients with Gorlin-Goltz syndrome, albinism, *Xeroderma pigmentosum,* and incomplete electronic medical records were not included in the analysis. Skin phototype was evaluated according to the Fitzpatrick scale (14).

Subjects were divided into immunosuppressed patients (IPs) and non-immunosuppressed patients (NIPs) upon their second diagnosis (comorbidity), if any. Solid and hematogenous malignancy history in the last 5 years, chronic viral infections, chronic kidney disease (CKD), diabetes mellitus (DM) (type I and II), asplenia, end-stage heart failure, and treatment with antirheumatic immunosuppressive drugs (including biologics and long-term systemic steroids) were considered immunosuppressive comorbidities.

The anatomical BCC sites were classified as follows: nose, forehead, cheeks, neck, ears, scalp, trunk, and extremities. The neck and face were highlighted as photo-exposed areas and the trunk and extremities as regions with intermittent sun exposure. BCC size was divided into three subgroups: small (<10 mm), medium (10–30 mm) and large (>30 mm). The BCC specimens were analyzed by an expert in dermatopathology and the classification was made upon the 2018 World Health Organization (WHO) guidelines (15). BCCs were divided into low-risk and high-risk recurrence subtypes based on their pathology features.

### 2.3 Statistical analysis

All investigative data were collected into a central database (Microsoft Excel). The statistical analysis was completed using the R software. Descriptive and graphical analysis was used to check assumptions of normality and linearity for all study variables. Clinical and demographic characteristics were compared between patients with BCC who had immunosuppressed comorbidities and those who did not.

The normality of the distributions was tested rigorously by the Shapiro–Wilk Test and the symmetry of the non-normal distributions was analyzed by looking at the skewness and kurtosis indicators. Regarding the age of the patients from both groups, the *p*-value is greater than 0.05, therefore, the distribution of the given data is not different from the normal distribution significantly. Moreover, by testing the normality of the data concerning the size of the carcinoma, it results that our data is not distributed normally (*p* < <0.001).

Differences between various subgroups were analyzed using an unpaired two-tailed *t*-test or Mann–Whitney *U* test for all continuous scale data between subgroups, where appropriate. Chi-square test (*χ*^2^) with/ without Yates’ continuity correction, Fisher’s exact Test, or Fisher’s exact test with simulated *p*-value (based on 1 × 10^8^ replicates) were used, where applicable. They were also used in the subgroup analysis, where we assessed whether there were statistical differences between IPs and NIPs concerning sex, skin type, site of lesion, tumor size (<10 mm, 10–30 mm, ≥30 mm), and age groups (30–54, 55–74, >75). The Chi-Square Goodness fit Test was used to study if the distribution of comorbidities among IPs is uniform while proportions tests were used to examine the prevalence of a particular group/feature. Linear relations with a *p*-value (two-sided) ≤0.05 were considered significant.

## 3 Results

This study included 275 patients with 292 BCCs, as follows: 262 patients had one BCC, 11 patients had two BCCs and 2 patients had four BCCs. The patients were divided by sex into 142 female and 133 male patients, with a F/M ratio equal to 1.067. Most of the patients (64.73%) had various secondary health conditions (*p* < 0.001) and among them, IPs represented 80.34% (*p* < 0.001). Gender distribution, living environment, and skin phototypes did not vary between IPs and NIPs. The average age of IPs (67.88) is higher than the average age of NIPs (62.38). IPs were more likely to present multiple BCCs (*p* = 0.02706, Fisher’s Exact Test). Table 1 displays the demographic characteristics of patients.

**Table 1:** Demographic characteristics of patients.

Total (*N* = 275)			NIPs	IPs	*p*-value
			132 (48%)	143 (52%)	0.5465^a^
Age, mean (SD)		65.24 (0.70)	62.38 (1.12)	67.88 (0.80)	<0.001^b^
Age group, *n* (%)	31–54	42 (15.27%)	32 (24.24%)	10 (6.99%)	<0.001^c^
	55–74	171 (62.18%)	79 (59.85%)	92 (64.34%)	
	>75	62 (22.55%)	21 (15.91%)	41 (28.67%)	
Gender, *n* (%)	M	133 (48.36%)	65 (49.24%)	68 (47.55%)	0.8733^c^
	F	142 (51.64%)	67 (50.76%)	75 (52.45%)	
Skin type, n (%)	2	142 (51.64%)	66 (46.48%)	76 (53.52%)	0.6885^c^
	3	133 (48.36%)	66 (49.62%)	67 (50.38%)	
Living environment, n (%)	Rural	138 (50.18%)	65 (49.24%)	73 (51.05%)	0.8582^c^
	Urban	137 (49.82%)	67 (50.76%)	70 (48.95%)	

IP, immunosuppressed patient; NIP, non-immunosuppressed patient.

a Proportion test.

b *t*-test.

c Chi-square test.

The distribution of comorbidities among IPs (Table 2) reveals a non-uniform distribution (*p* < 0.001), DM (type II) being the most frequent comorbidity and representing almost 50% of the IPs. Patients undergoing immunosuppressive therapies were treated for arthritis (62.5%) and lupus erythematosus (38.5%) with oral steroids and immunosuppressant regimens. Patients with chronic infections had a history of hepatitis viruses (62.5%) and pulmonary tuberculosis (37.5%).

**Table 2:** Comorbidities, living environment, and gender distributions among IPs.

Comorbidity	Living environment		Gender		Total, *n* (%)
	Rural, *n* (%)	Urban, *n* (%)	F, *n* (%)	M, *n* (%)	
Cancer history	16 (42.11%)	22 (57.89%)	25 (65.79%)	13 (34.21%)	38 (26.57%)
Immunosuppressants	6 (50.00%)	6 (50.00%)	12 (100%)	—	12 (8.39%)
DM	37 (54.41%)	31 (45.59%)	32 (47.06%)	36 (52.94%)	68 (47.55%)
CKD	9 (75.00%)	3 (25.00%)	4 (33.33%)	8 (66.67%)	12 (8.39%)
Chronic infections	5 (38.46%)	8 (61.54%)	2 (15.38%)	11 (84.62%)	13 (9.09%)
	*p* = 0.2799^a^		*p* < 0.001^a^		*p* < 0.001^b^

CKD, chronic kidney disease; DM, diabetes mellitus.

a Fisher exact test.

b Chi-square-goodness-fit.

**Figure 1:**
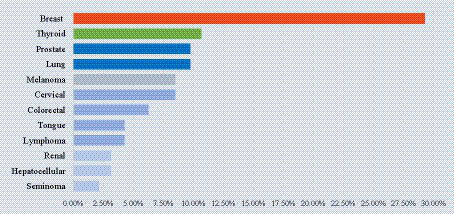
Distribution of cancer history among BCC patients. Breast, thyroid, prostate, and lung cancer represented the most encountered malignancies.

Patients with a cancer history represented almost 30% of IPs. Among these, breast cancer was the most prevalent (29.27%), followed by thyroid cancer (10.64%), lung cancer (9.76%) and prostate cancer (9.76%) (Figure 1). None of the patients had an HIV infection, asplenia, end-stage heart failure, or primary immunodeficiencies, nor did they undergo organ transplantation. All patients with CKD had a pre-dialysis stage. IPs comorbidities did not vary with respect to the living environments. In regards to the gender of the patients, we found that female patients with BCCs more frequently presented a cancer history or underwent immunosuppressive therapies than men (Table 2).

Sample sizes did not vary among IPs (*p* = 0.0958, Fisher exact test) (see Table 3). BCC subtypes were evaluated among IPs (*p* = 0.0064, Fisher Exact Test with simulated *p*-value) as shown in Table 4. Nodular and superficial subtypes were the most frequent subtypes in patients with a history of cancer and DM patients. Patients with CKD presented superficial and ulcerated BCCs in equally high proportions. The superficial subtype remained the most prevalent for each group.

**Table 3:** Specimen characteristics in IPs and NIPs.

		Total (*N* = 292)	BCCs in NIPs (*N* = 134)	BCCs in IPs (*N* = 158)	*p*-value
Size (mm), mean (SD)		14.55 (11.15)	14.44 (10.90)	14.65 (11.39)	0.2577^a^
Size group (mm)	<10	92 (31.51%)	49 (36.57%)	43 (27.22%)	0.0806^b^
	10–30	175 (59.93%)	71 (52.98%)	104 (65.82%)	
	>30	25 (8.56%)	14 (10.45%)	11 (6.96%)	
Subtypes	Superficial	124 (42.46%)	59 (44.03%)	65 (41.14%)	0.1727^c^
	Nodular	58 (19.86%)	23 (17.16%)	35 (22.15%)	
	Ulcerated	47 (16.10%)	19 (14.18%)	28 (17.72%)	
	Pigmented	7 (2.40%)	1 (0.75%)	6 (3.80%)	
	Infiltrative	27 (9.25%)	14 (10.45%)	13 (8.23%)	
	Others	29 (9.93%)	18 (13.43%)	11 (6.96%)	
Localization area	Intermittently/not sun-exposed areas	119 (40.75%)	57 (42.54%)	62 (39.24%)	0.6514^b^
	Photo-exposed areas	173 (59.25%)	77 (57.46%)	96 (60.76%)	
Localization	Trunk	105 (35.96%)	47 (35.07%)	58 (36.71%)	0.3328^d^
	Limbs	14 (4.79%)	10 (7.46%)	4 (2.53%)	
	Nose	66 (22.61%)	31 (23.14%)	35 (22.15%)	
	Cheeks	54 (18.49%)	20 (14.93%)	34 (21.52%)	
	Frontotemporal	28 (9.59%)	15 (11.19%)	13 (8.23%)	
	Ears	7 (2.40%)	3 (2.24%)	4 (2.53%)	
	Scalp	10 (3.42%)	3 (2.24%)	7 (4.43%)	
	Neck	8 (2.74%)	5 (3.73%)	3 (1.90%)	

IP, immunosuppressed patient; NIP, non-immunosuppressed patient.

a Mann–Whitney test.

b Chi-square test.

c Fisher exact test.

d Fisher exact test with simulated *p*-value.

**Table 4:** BCC subtypes distribution.

BCC subtype	*n* (%)				
	Cancer history	Immunosuppressants	DM	CKD	Chronic infections
Infiltrative	—	1 (6.25%)	10 (14.71%)	1 (7.69%)	1 (7.14%)
Nodular	16 (34.04%)	1 (6.25%)	16 (23.53%)	2 (15.38%)	—
Pigmented	1 (2.13%)	—	5 (7.35%)	—	—
Superficial	17 (36.17%)	10 (62.5%)	22 (32.35%)	5 (38.46%)	11 (78.57%)
Ulcerated	10 (21.28%)	1 (6.25%)	11 (16.18%)	5 (38.46%)	1 (7.14%)
Other	3 (6.38%)	3 (18.75%)	4 (5.88%)	—	1 (7.14%)

CKD, chronic kidney disease; DM, diabetes mellitus.

BCC subtypes also vary between patients with a history of cancer and the other IPs (*p* = 0.0308), as nodular and superficial subtypes are most relevant to this subset of patients (34.04 and 36.17%, respectively). Low-risk and high-risk histopathological subtypes did not vary between IPs and NIPs (*p* = 0.4269) nor among IPs (*p* = 0.5819).

A significant difference was found between BCC localizations in IPs, as shown in Table 5. Patients with chronic infections and DM most often had trunk BCCs and nose BCCs, while patients with CKD and following immunosuppressants most frequently presented truncal BCCs and cheeks BCCs. The nose was the most prevalent localization for cancer history patients, followed by the trunk.

**Table 5:** BCC localizations distributions among subgroups, *n* (%).

Localization	Cancer history	Immunosuppressants	DM	CKD	Chronic infections
Nose	14 (29.79%)	—	15 (22.06%)	1 (7.69%)	5 (35.71%)
Forehead	7 (14.89%)	2 (12.5%)	2 (2.94%)	2 (15.38%)	—
Cheeks	10 (21.28%)	4 (25%)	15 (22.06%)	4 (30.77%)	1 (7.14%)
Neck	—	1 (6.25%)	1 (1.47%)	—	1 (7.14%)
Ears	2 (4.26%)	1 (6.25%)	1 (1.47%)	—	—
Scalp	—	—	7 (10.29%)	—	—
Trunk	11 (23.40%)	8 (50%)	27 (39.71%)	5 (38.46%)	7 (50%)
Limbs	3 (6.38%)	—	—	1 (7.69%)	—

CKD, chronic kidney disease; DM, diabetes mellitus. *p*-value = 0.008063, Fisher exact test with a simulated *p*-value.

## 4 Discussion

Basal cell carcinoma is a skin cancer that arises from the basal keratinocytes. Its incidence rate increases with age (16). Most of the patients were between 55–74 years old, in line with the literature (17). More than 66% of BCC patients presented a secondary diagnosis (comorbidity). This finding can be explained by the majority of patients older than 55 years.

**Figure 2:**
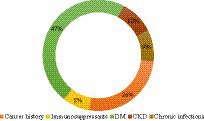
Comorbidities distribution among IPs. Diabetes mellitus type II was found in almost half of the IPs, a history of cancer in a quarter of the IPs, and each of the other three categories in approximately a tenth of the IPs. DM, diabetes mellitus; CKD, chronic kidney disease.

Interestingly, more than 80% of the BCC patients’ comorbidities represented an immune-impairing disorder and were regarded as immunosuppressive comorbidities (Figure 2). Multiple BCCs (epitheliomatosis) were more frequent in IPs. This last finding was previously attributed to SCC (11), but not to BCC.

The medium BCC size (1.5 cm) did not vary significantly between IPs and NIPs, and BCCs between 10 and 30 mm were the most common in both groups. A study that compared clinicopathologic characteristics of BCC (including size) in 69 immunosuppressed renal transplant recipients (RTCs) did not find any differences from non-RTCs (18). Among IPs and NIPs, the superficial BCC was the most frequent, followed by nodular and ulcerated BCC. Additionally, as BCC usually arises in photo-exposed areas (2, 16), almost 60% of all specimens were distributed in these areas.

Diabetes mellitus represents a public health problem worldwide, with an incidence of 11.6% in Eastern Europe (19). It was the most frequent comorbidity among IPs (Figure 2). This observation is consistent with a study that revealed a high association between DM and BCC. Hyperglycemia and hyperinsulinemia were proposed as possible carcinogenesis factors, via reactive oxygen species (ROS) (20).

Nearly a quarter of IPs had a history of cancer. Likely, the neoplastic microenvironment may promote BCC carcinogenesis by inducing immune tolerance, recruitment of regulatory T cells, and suppression of NK cells (21, 22).

**Figure 3:**
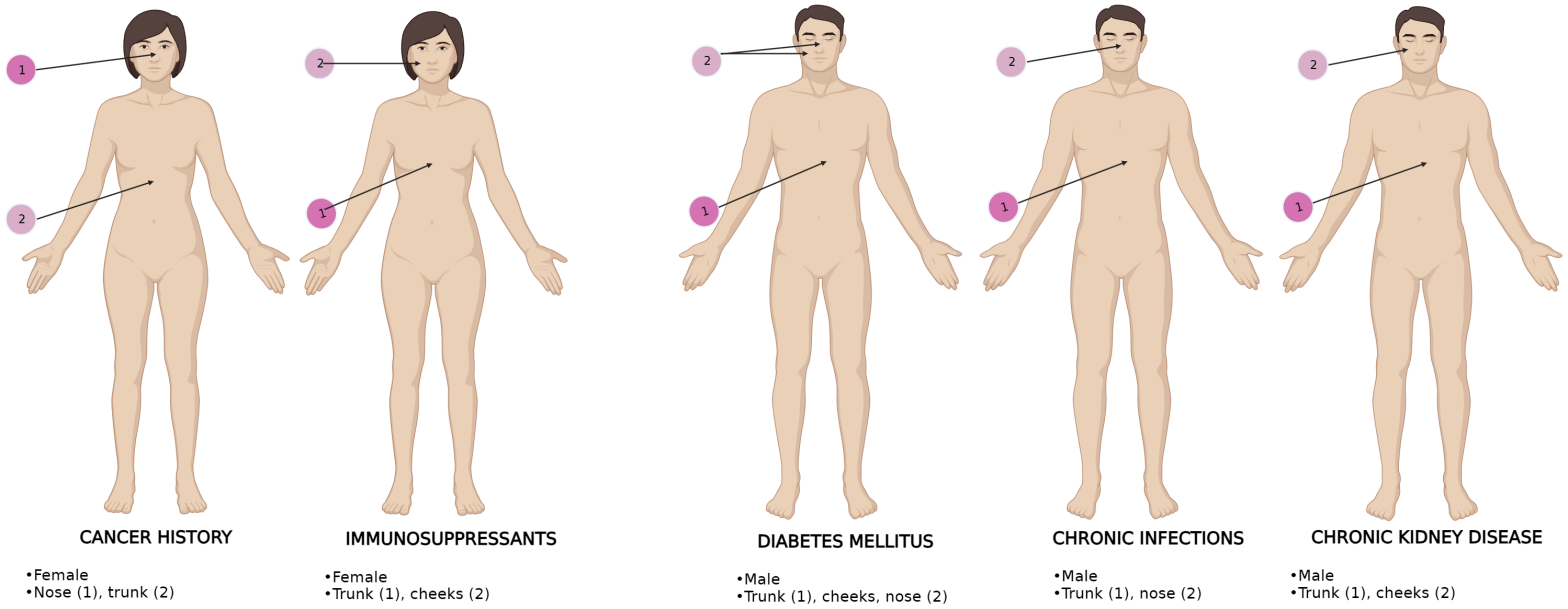
BCC anatomical distribution upon gender and comorbidities in IPs. The most frequent (1) and second most frequent (2) localizations are displayed. BCC localizations are represented for each comorbidity. The most representative gender is shown for each comorbidity.

As regards BCC dimensions, they did not vary significantly among IPs, but the gender distributions, histopathological subtypes, and anatomical localizations (Figure 3) were different among IPs. Although the superficial subtype remained the most prevalent, the ulcerated subtype was equally high in patients with CKD. Patients with pre-dialysis CKD have a high risk of developing BCC (18). We presume high ulceration rates may be secondary to increased oxidative stress in CKD patients, who present high levels of chlorinated, nitrosative, and carbonyl stress, alongside the usual ROS (23). Additionally, the same particular neoplastic microenvironment (21) may be responsible for different pathology specimens in this subset of patients compared to all the other IPs.

## 5 Strengths and limitations

The large number of participants is a strength of this study. Unlike most studies, OTRs were not encountered in this BCC population and novel results regarding BCC characteristics in non-OTRs were obtained. Additionally, the correlations between BCC localizations and secondary immunodeficiencies represent a potential clinical innovation and may be particularly useful in daily practice. Finally, this study has its limitations as well. Since data were not collected in a predefined proforma, the precise interval between BCC appearance in healthy skin and comorbidities diagnoses was not available. The retrospective, observational analysis represents a limitation of the study.

## 6 Conclusion

Patients with BCC predominantly have immunosuppressive comorbidities. Immunosuppressed patients do not develop aggressive BCC subtypes and BCC does not arise earlier in their life, unlike patients with cSCC. BCC on the nasal pyramid is frequent in patients with a history of cancer. The ulcerated form of BCC is more confined to patients with chronic kidney disease. BCCs in patients with secondary immunodeficiencies contrasted with immunocompetent patients regarding gender, histopathological subtype, and localizations. Multiple BCCs were more frequent in patients with secondary immunodeficiencies. Hence, we consider BCC a clinical indicator of immunodeficiency.

Prospective studies regarding the spectrum of BCC in cohorts with neoplastic history and chronic kidney disease are necessary. Understanding the relationships between basal cell carcinoma and solid or hematogenous cancers may be the promoter of early cancer screening campaigns in BCC patients.

## Data availability statement

The raw data supporting the conclusions of this article will be made available by the authors, without undue reservation.

## Ethics statement

The studies involving humans were approved by Colentina Clinical Hospital Board. The studies were conducted in accordance with the local legislation and institutional requirements. The ethics committee/institutional review board waived the requirement of written informed consent for participation from the participants or the participants’ legal guardians/next of kin because the study was retrospective and non-interventional. Data were collected from an electronic database.

## Author contributions

LS: Conceptualization, Data curation, Investigation, Methodology, Visualization, Writing – original draft, Writing – review & editing. MPe: Formal analysis, Methodology, Software, Writing – original draft. FS: Data curation, Formal analysis, Writing – original draft. AS: Data curation, Formal analysis, Writing – original draft. MPo: Visualization, Writing – original draft, Data curation. OS: Conceptualization, Methodology, Supervision, Validation, Visualization, Writing – review & editing.
